# Erythropoietin and organ protection: lessons from negative clinical trials

**DOI:** 10.1186/s13054-014-0526-9

**Published:** 2014-09-11

**Authors:** Ronald G Pearl

**Affiliations:** Department of Anesthesiology, Perioperative and Pain Medicine, Stanford University School of Medicine, 300 Pasteur Drive, Room H3589, Stanford, CA 94305-5640 USA

## Abstract

Based on its pleiotropic effects, erythropoietin can decrease inflammation, oxidative stress, and apoptosis. Erythropoietin provides organ protection for the heart, brain, and kidney in diverse preclinical animal studies, especially models that include ischemia–reperfusion injury and/or inflammation. However, large clinical studies in coronary reperfusion, heart failure, stroke, acute kidney injury, and chronic renal disease have failed to demonstrate improved outcomes. A study in a previous issue of *Critical Care* examining the ability of erythropoietin to prevent or ameliorate acute kidney injury in patients undergoing complex valvular heart surgery is similarly negative. The failure of erythropoietin in clinical studies may be due to an inadequate dose, since the receptors responsible for organ protection may require higher concentrations than those responsible for erythropoiesis. However, as has occurred in studies in sepsis and acute respiratory distress syndrome, the negative studies probably reflect an inadequate understanding of the complexity of the underlying processes with multiple redundant and interacting pathways that may differ among the large number of different cell types involved. As tools to understand this complexity and integrate it on an organismal basis continue to evolve, we will develop the ability to use erythropoietin and related nonhematopoietic agents for organ protection.

In a previous issue of *Critical Care*, Kim and colleagues report a randomized double-blind study of the effects of erythropoietin on acute kidney injury (AKI) in patients undergoing complex valvular cardiac surgery [1]. AKI occurred in 34% of patients, but there were no differences between the two groups in the incidence or severity of AKI, increases in AKI biomarkers (cystatin C and neutrophil gelatinase-associated lipocalin) or increases in markers of inflammation (interleukin-6 and myeloperoxidase). Despite strong preclinical data and a previous small clinical trial, this was a negative study. An understanding of the rationale for using erythropoietin for organ protection and a review of negative clinical trials can help us understand the information necessary for future studies.

Erythropoietin was first identified as the major regulator of erythropoiesis. The molecule was isolated in 1977, and the gene was cloned in 1985. Recombinant human erythropoietin was approved in the United States in 1989 for treatment of anemia associated with chronic renal failure, and its use subsequently expanded to additional indications. Erythropoietin is a 30.4 kDa glycoprotein with 165 amino acids, and is a class I cytokine predominantly produced by peritubular interstitial fibroblasts in the renal cortex and outer medulla. The majority of physiological effects are due to binding to erythropoietin receptors that are found on multiple cell types, including renal tubular and collecting duct cells, neurons, astrocytes, microglia, cardiomyocytes, and vascular cells [2]. Following binding of erythropoietin, the erythropoietin receptor activates JAK2, a member of the Janus-type protein tyrosine kinase family, which then activates multiple signaling pathways including mitogen-activated protein kinase, phosphatidylinositol 3-kinase, signal transducer and activator of transcription 5, and protein kinase B. Different signaling pathways may be activated in different cell types. Erythropoietin has pleiotropic cellular protective effects and can decrease inflammation, oxidative stress, and apoptosis.

Erythropoietin has been studied for brain, heart, and kidney protection [2]. Erythropoietin improves survival in neurons during hypoxia in cell culture, animal studies demonstrate protection against ischemia, and initial clinical trials suggested protection in ischemic stroke, but larger trials did not confirm benefits and even suggested adverse outcomes [3]. For the heart, animal studies demonstrate protection after ischemia–reperfusion, and small clinical studies demonstrated benefit in the setting of percutaneous coronary intervention for myocardial infarction. However, large clinical trials did not demonstrate benefits [4,5]. Similarly, trials in patients with anemia and heart failure do not demonstrate improved outcomes and raise concerns about increased complications [6,7]. Aggressive use of erythropoietin for treatment of anemia in patients with renal disease or malignancy has also failed to demonstrate benefits [8–10].

The story of erythropoietin use for renal protection has been similar. Animal data demonstrate protective effects, especially in the setting of ischemia–reperfusion injury [2]. However, clinical studies in renal transplantation and chronic kidney disease have been negative [11]. AKI occurs in response to renal ischemia and inflammation and increases morbidity, hospital length of stay, and short-term and long-term mortality. AKI occurs in approximately one-half of patients undergoing cardiac surgery. The anti-oxidant, anti-inflammatory, and anti-apoptotic effects of erythropoietin suggest it may be effective in preventing or ameliorating AKI in this setting. In a pilot study by Oh and colleagues, erythropoietin decreased AKI and mortality in patients undergoing coronary artery bypass surgery [12]. However, in the study by Kim and colleagues and in another large cardiac surgery study [13], erythropoietin did not decrease the incidence of AKI, did not improve outcome, and did not decrease markers of inflammation. These negative data are consistent with the EARLYARF trial where erythropoietin did not prevent or ameliorate the course of AKI in ICU patients with elevated urinary biomarkers for renal injury [14].

Erythropoietin may have failed in clinical trials due to insufficient dose. Preclinical studies have frequently used doses in the range of 1,000 to 5,000 IU/kg, an order of magnitude higher than the dose of 300 IU/kg used in many clinical studies. Although this lower dose is adequate for erythropoiesis, the erythropoietin receptors responsible for organ protection are different. Higher doses of erythropoietin could be used but may have adverse effects such as thrombosis and hypertension. One future approach may be the use of nonhematopoietic derivatives such as carbamylated erythropoietin that produce cellular and organ protection in animal studies.

Although dose may be an issue, the multiple negative clinical studies suggest that our approach and understanding of renal, cardiac, and neurologic problems is too simplistic. There is a complex interplay between vascular abnormalities and inflammatory mediators, there are a myriad of different cell types involved, and, within each individual cell, there are complex interactions among multiple pathways that each have redundancy. In this setting (and similarly in sepsis and acute respiratory distress syndrome), the net impact of an intervention that itself has pleiotropic effects becomes unpredictable, resulting in negative clinical trials. For example, erythropoietin is considered to be anti-inflammatory but can worsen inflammation-induced injury in other studies [15,16]. Effective use of erythropoietin for organ protection will require a better understanding of the relevant processes that occur at the cellular level [17] and the ability to phenotype individual patients for relevant factors that may affect outcome. With increased knowledge, erythropoietin may become an important weapon for preventing AKI, but it will not be a magic bullet.

## Abbreviation

AKI: Acute kidney injury
